# An exceptionally high wave at the CNR-ISMAR oceanographic tower in the Northern Adriatic Sea

**DOI:** 10.1038/s41597-021-00825-x

**Published:** 2021-01-29

**Authors:** Luigi Cavaleri, Francesco Barbariol, Mauro Bastianini, Alvise Benetazzo, Luciana Bertotti, Angela Pomaro

**Affiliations:** Consiglio Nazionale delle Ricerche, Istituto di Scienze Marine (CNR-ISMAR), Venice, Italy

**Keywords:** Civil engineering, Physical oceanography

## Abstract

On December 15, 2009, a very high wave crest was recorded by a local camera at the CNR-ISMAR oceanographic tower, 15 km offshore Venice in the Northern Adriatic Sea (Italy). The height of the estimated crest elevation appears well beyond the value (1,25·*H*_*s*_) commonly used to identify a wave as freak. We document the wave event with a full description of the corresponding met-ocean conditions and related measurements, of which we provide a critical analysis.

## Abstract (summary)

**Table:** 

Measurement(s)	atmospheric wind direction • atmospheric wind speed • wave
Technology Type(s)	weather station • acoustic AWAC system • waverider buoy • Camera Device
Sample Characteristic - Environment	coastal sea water
Sample Characteristic - Location	Adriatic Sea

Machine-accessible metadata file describing the reported data: 10.6084/m9.figshare.13415780

## Background & Summary

**Fig. 1:**
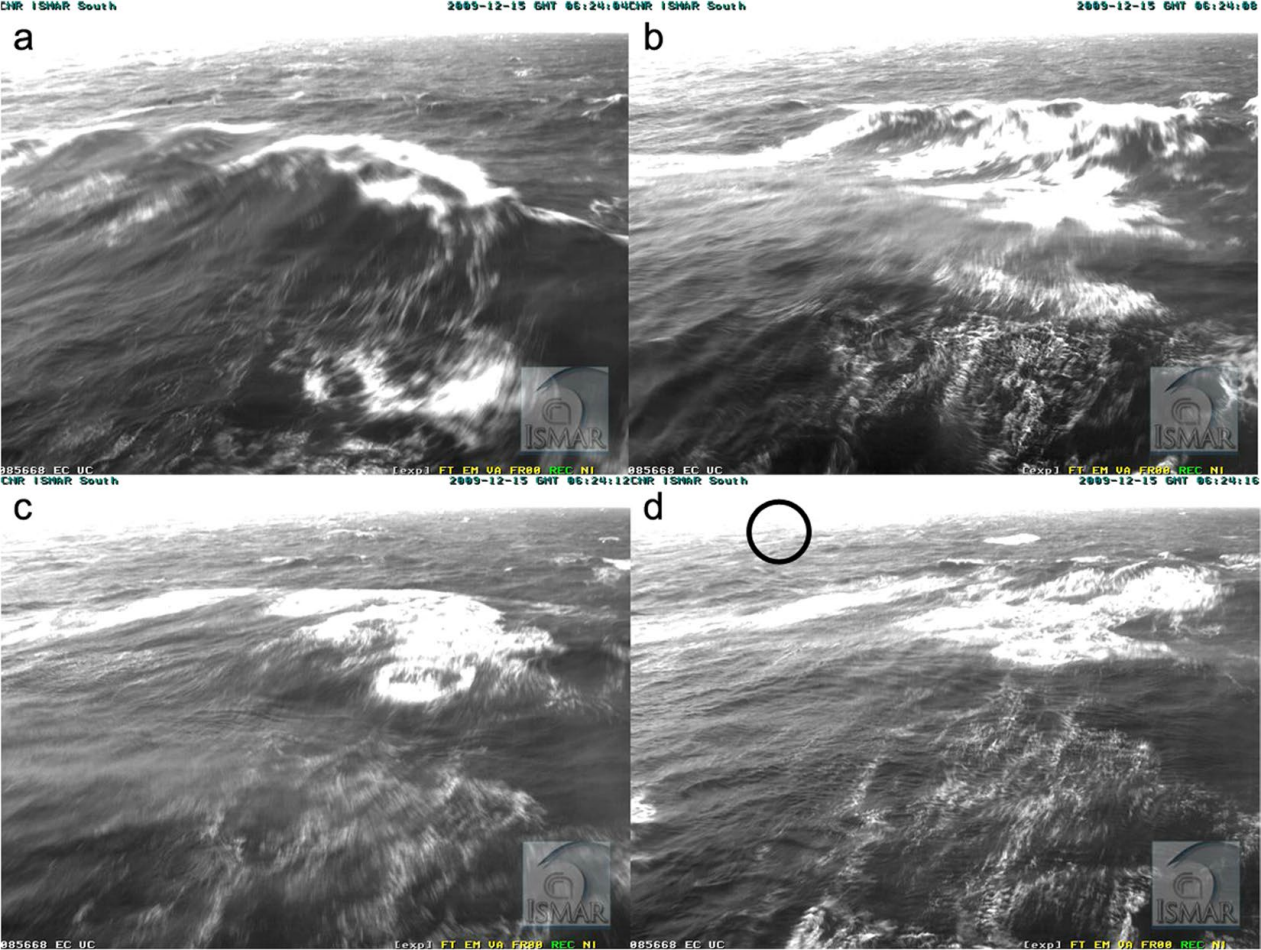
Frames from the onboard webcam. 15 December 2009, 07.24 am local time. From (**a**) to (**d**) frames at four second intervals. The black circle shows the position of the buoy.

The Institute of Marine Sciences of the National Research Council of Italy (CNR-ISMAR, henceforth ISMAR) manages an oceanographic tower in the Northern Adriatic Sea^1^. Beside an array of meteorological and oceanographic instruments, three web-cameras monitor the situation of the surrounding sea and underwater conditions. On December 15, 2009 the South-looking camera documented at 0.5 Hz the passing of a very high wave crest to be then followed in the sequential images for further 20 sec or so. The relevant recorded frames are shown in Fig. 1. The full sequence is available at PANGAEA. Here we analyze the event and the available information. In the Methods section we describe in detail the procedure followed for the quantification of the crest height. This is then followed (Technical Validation) by a description of the meteo-oceanographic situation together with a critical analysis of the approximations implied in our estimates. Finally, we summarize the data and its characteristics.

In the last few years freak waves have lost part of their appeal. Once considered literally “freak events”, experience and theory^2,3^ have shown them to be part of the daily world. The use of the stereo system, particularly from the CNR-ISMAR oceanographic tower, here available only after 2009, and the work by, among others^4,5^, have shown that freak events are the norm in a storm. It was only the tradition of single point measurements that made them to appear rare and freak. However, the reported event appears to be out of the reasonably expected range.

## Methods

In this section we describe the perspective analysis that has been performed with reference to the available data from different sources.

**Fig. 2:**
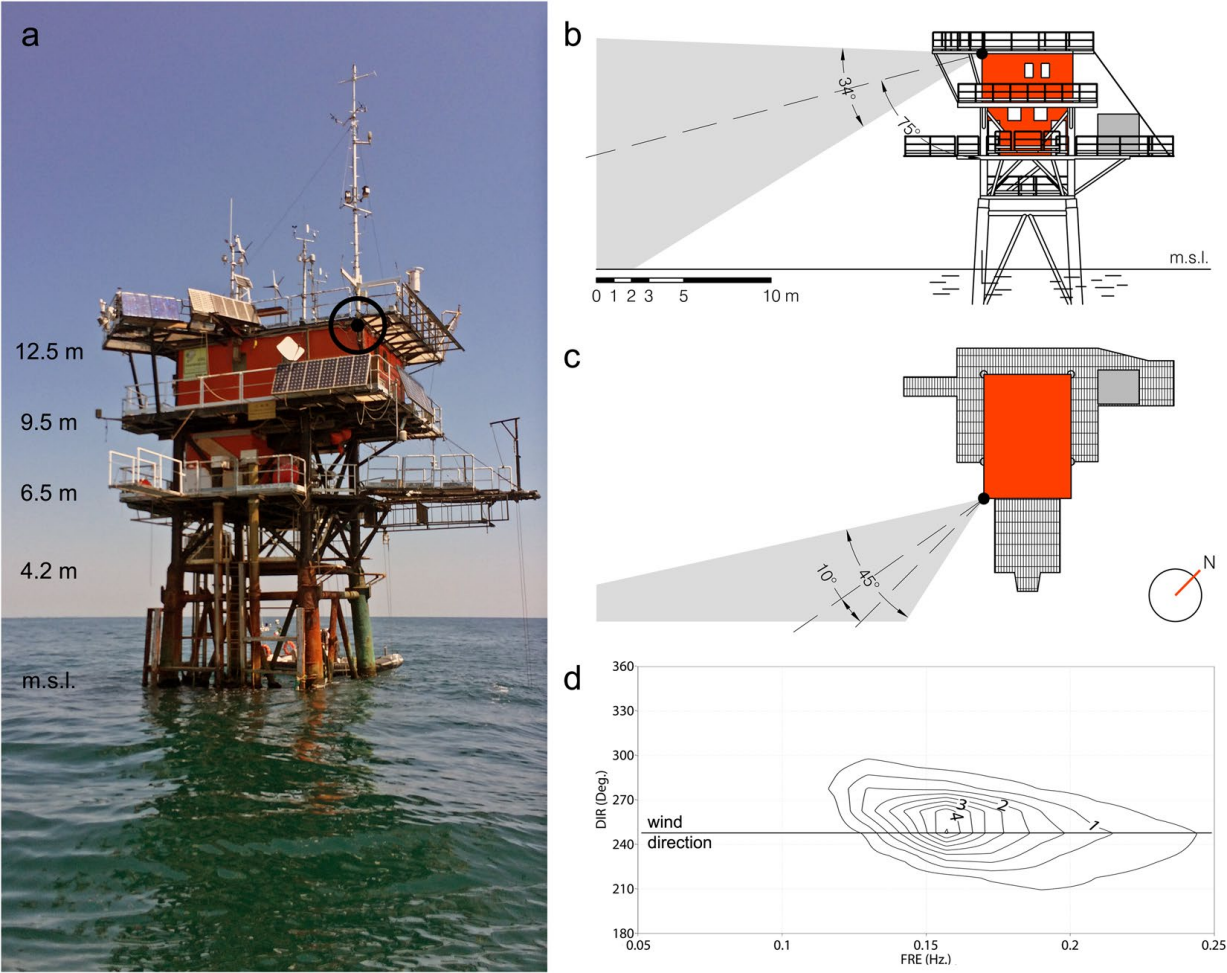
(**a**) The ISMAR oceanographic tower. The circle indicates the webcam position at 12.5 m height. (**b**) Vertical view angle of the webcam. (**c**) Horizontal view angle of the webcam. The large dot shows the ADCP position. The arrow head points to the outside position of the Waverider buoy, 120 m from the tower. (**d**) 2D (f,θ) wave spectrum at the time of the event. The line indicates the wind flow direction.

The camera that recorded the freak wave event images was mounted at a fixed position, so it was possible to estimate a posteriori the position and the geometry of the framed portion of the sea surface elevation field. The focus has been on the wave crest height because the previous trough is not visible. The webcam was a Mobotix M12 with installation height 12 m, baseline 20 m, focal length 8 mm (the equivalent of a 35 mm camera), horizontal and vertical image angles 45° and 34°, frames saved at 2 second intervals. Its geometrical position and view angles are shown in Fig. 2, panels b and c. Basically, the sea surface was visible from 20 m distance till the horizon. Obviously, given its height and as seen in the first ones of the auxiliary frames, the crest is visible before this distance. The light was scarce, 6.24 UTC (7.24 am local time with sunrise at 7.43 am), with a cloudy sky, nevertheless the system had enough sensitivity to document the situation.

**Fig. 3:**
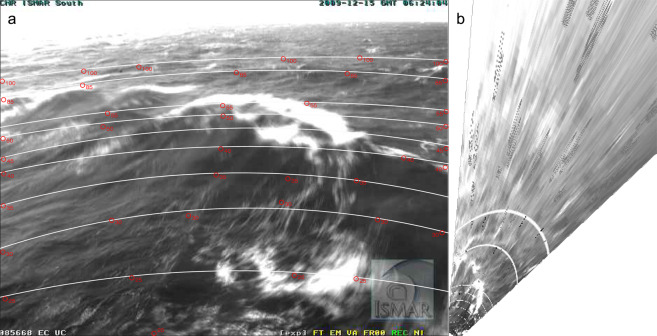
(**a**) (same frame as in panel 1a). Isolines of various distances (m) from the vertical of the webcam position (see Fig. 2). Maximum distance line at 100 m. (**b**) shows the planar, undistorted view of the same field.

Two approaches have been used to estimate the maximum crest height, both aiming at determining the vertical displacement of the crest with respect to the mean sea level. In the first one, the images recorded by the camera were rectified through a homographic transformation relating the camera plane with the horizontal mean sea plane. This allows a recovery of the sea plane xy-axes and, consequently, an alignment of the image data with the geographical axes (the one orthogonal to the plane is vertical). After the image calibration procedure, necessary to estimate the focal length, the optical center and the distortion of the camera lens, the parameters of the homography matrix were determined using points of known 2-D coordinates located on the mean sea plane. In this respect, thirty-four positions were considered spanning the entire image plane, which were then used to produce the rectified image (Fig. 3, panel b). To this end, the TriDmetriX® software was used. After rectification, the key point was to determine for each frame the horizontal xy-position of the maximum crest elevation, from which to intersect the inclined optical axis coming from the camera plane, so to obtain the vertical displacement of the wave crest. On the rectified image, that position was specified tracking along the crest direction a segment connecting the two wave troughs on either side of the crest. This segment turned out about 50 m long, a length close to the expected one for waves having a 5.8 s peak period, hence confirming, a posteriori, the meaningfulness of the procedure. The mid-position of the segment was considered representative of the crest location, that is the crest was located, in the rectified images, midway the lateral troughs. Following this approach, the crest maximum elevation was estimated at 6.4 m above the mean sea level. However trivial, tidal level was taken into account.

**Fig. 4:**
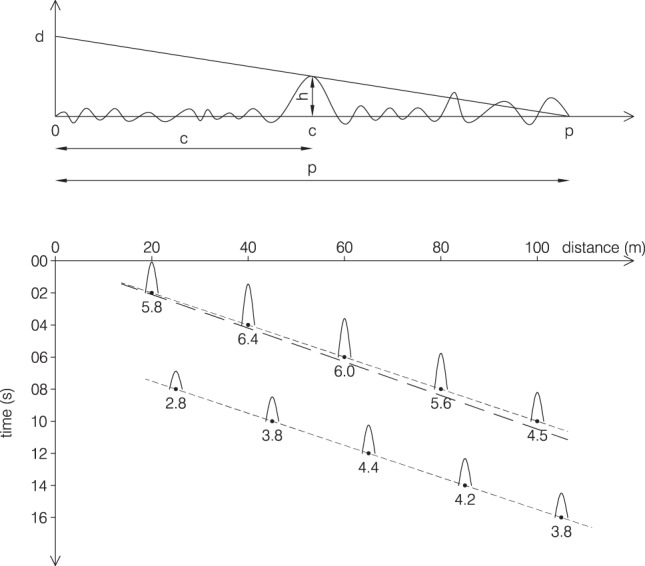
(**a**) Essential geometry for the derivation of the crest height. d is the height of the webcam (see Fig. 2). (**b**) Crest height, in space and time, of the two sequential waves seen in Fig. 1. Full sequence at^7^. The long dash line represents the corresponding group motion.

In the second approach we used a more direct evaluation procedure. A boat at known (taught rope) distance moved along circle sections, each at progressively larger distances (till about 150 m). A dense sequence of positions of the boat were marked on the screen displaying the webcam view, while recording the corresponding boat coordinates. The procedure was repeated three times. This provided a sequence of isolines similar to the ones displayed in Fig. 3a. The isoline systems derived with the two different methods turned out very similar. As an example the 100 m distance isoline derived with the second method is shown in the panel as a dashed line. In each image the distance of the crest was obtained a) using the short crestedness of the wave and deriving the distance of its two side extremes, b) connecting these two points with a straight line (taking the optics of the image into account), c) deriving the distance of the position under the crest, d) taking into account the distance of the (relatively far) sea surface position seen along the webcam-crest perspective. The trivial geometry is reported in Fig. 4, panel a. We will then discuss the implied approximations.

Figure 4, lower panel provides the sequence of estimated crest heights, in time and space. Not yet mentioned, a second, less macroscopic crest was following the first one, somehow taking over in time, albeit at lower level, its role. This suggests that these waves could be part of a group whose propagation is shown by the heavier dash line.

### Custom code

The first operation which is required for the determination of the parameters of the homography is the camera calibration phase, that enables to evaluate the radial distortion correction data A1, A2 and R0 of the camera. In order to do this, we used the Camera Calibration Toolbox for Matlab.

The technique requires the camera to observe a reference target (chessboard) of known pattern and size placed with different orientations in front of the camera. This is the standard operation to get reliable internal parameters of the camera, which have been obtained using the above mentioned Matlab toolbox.

The algorithm provides images acquisition and requires the operator to extract the grid corners. After this operation is done, it shows the boundary of the calibration grid. The program automatically counts the number of squares in both dimensions, and shows the predicted grid corners in absence of distortion. The corners are extracted to an accuracy of about 0.1 pixel.

After corner extraction, the code automatically generates a Matlab data file containing all the information gathered throughout the corner extraction stage (image coordinates, corresponding 3D grid coordinates, grid sizes, etc.).

Hence, the routine “Calibration” of the above cited toolbox allows to run the main camera calibration procedure, which is performed in two steps: an initialization phase and a non-linear optimization phase.

The initialization step computes a closed-form solution for the calibration parameters not including any lens distortion.

The non-linear optimization step minimizes the total re-projection error (through the least squares method) over all the calibration parameters.

The list of internal parameters that are being calculated by the algorithm is the following:

- focal length (fc), expressed in pixels;
- principal point (cc);
- skew coefficient (alpha_c), which defines the angle between the x and y pixel axes;
- the image distortion coefficients (radial and tangential, kc).

The optimization is done by iterative gradient descent with an explicit (closed-form) computation of the Jacobian matrix.

Once the calibration has been computed, the routine “Undistort images” allows to generate the undistorted version of one or multiple images given the pre-computed intrinsic camera parameters.

Photogrammetric techniques are based on the so called “pin-hole” model. This model represents a synthetic geometrical scheme of the camera functioning. The elements that compose this system are: the planar surface where the image takes origin, the projection center, which is the point from where the light gets access, the principal point, which is located at the intersection of the ray of light which is perpendicular to the planar surface and the planar surface itself, and the focal distance, which is the distance between the projection center and the principal point.

By means of these quantities we are able to describe, from a geometrical point of view, the image on the planar surface, with the sole exception of the radial deformations introduced by the camera lenses on the light rays’ trajectory across themselves.

After the introduction of the distortions correction, we analyze then the image orientation, or the camera position relative to the “real world” system of reference. The solution of this problem can be pursued through a geometrical method or through an analytical method.

The operation consists in a biunivocal transformation between two planar spaces, by means of eight parameters to calculate which at least 4, not aligned, points coordinates on both spaces must be known. The transformation allows passing from the real system of reference to the image system and viceversa, thus obtaining a vectorial graphical reconstruction of the image objective.

The undistort freak wave image is then obtained by means of the TriDmetrix 2014 software, which operates the rectification given the specified parameters.

## Data Records

The data resulting from the analysis described in the previous section are available at the link provided at PANGAEA^6–11^. In particular, the available data consist of:

- Photos of an exceptionally high wave at the Acqua Alta Oceanographic Tower on December 15, 2009^7^.
- AWAC processed wave parameters of an exceptionally high wave at the Acqua Alta Oceanographic Tower on December 15, 2009^8^
- Echo Sounder processed wave parameters of an exceptionally high wave at the Acqua Alta Oceanographic Tower on December 15, 2009^9^
- Waverider buoy processed wave parameters and raw data of an exceptionally high wave at the Acqua Alta Oceanographic Tower on December 15, 2009^10^
- Wind parameters at the Acqua Alta Oceanographic Tower on December 15, 2009^11^

## Technical Validation

We provide here a short description of the area, and of the meteorological conditions at the time of the event, together with a critical analysis of our estimates.

### The area and the instrumental set-up

**Fig. 5:**
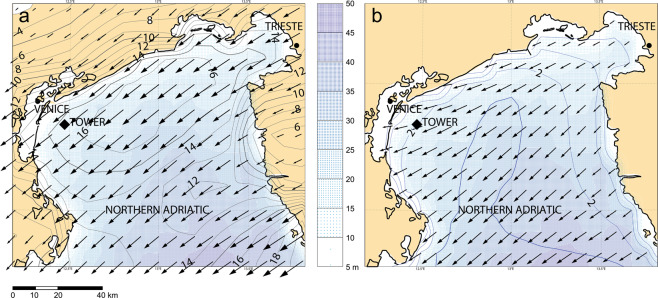
Northern Adriatic Sea. The colored shadowing provides the depth distribution (sea scale in meters). Isobaths are practically parallel to coast. (**a**) Arrows indicate the wind speed and direction. Isotachs at 2 ms-1 interval. (**b**) Wave field. Isolines of significant wave height at 0.5 meter intervals. The square indicates the tower position.

**Fig. 6:**
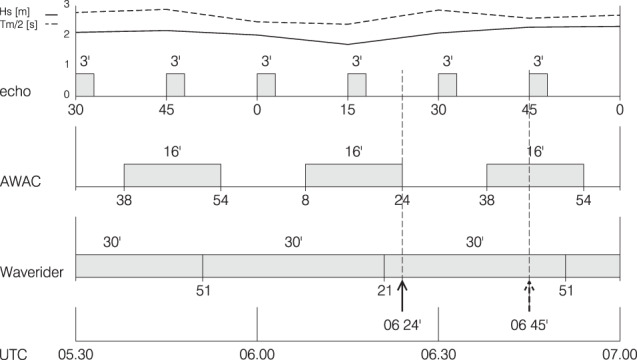
Timetable of data acquisition from the three wave measuring systems. The left vertical dash line and arrow point at the time of the freak wave event in Fig. 1. The right dash line shows when a second large wave was recorded. The upper lines represent the H_s_ and T_m_ values recorded by the echo-sounder system.

The Adriatic Sea, 700 km long, 200 km wide, is the elongated basin to the East of Italy. Its northern part, roughly 100 × 100 km^2^ wide and the one of interest for the present purpose, is shown in Fig. 5. The bottom, mostly sand and mud, slopes (1/1000) down towards South-East. The tower (see in panel 2a its picture at the time of the event - position marked in Fig. 5) is 15 km offshore, on 16 m depth. The isobaths are practically parallel to the coast. Fully equipped for meteorological and oceanographic measurements^3^, the tower hosted, at the time of the event, three independent wave measuring systems: a down-looking echo-sounder (hereinafter *E*), an acoustic AWAC (Nortek AS, hence *A*) located at the bottom 20 m east of the tower (see Fig. 2c), and a directional Waverider buoy (hereinafter *B*) 120 m away in the same direction. The timetable of data acquisition is provided in Fig. 6. Because the presence of the freak wave (bulk wave conditions were not particularly extreme) was realized only a posteriori, for all the instruments only the integrated parameters are available.

### Meteorological and wave conditions

The meteorological conditions (see Fig. 5a) were a 15 ms^−1^ wind from 60° N (cold northeasterly Bora wind) with an active generation, as documented by the numerous whitecaps visible in Fig. 1. The significant wave height measurement *H*_*s*_ provided by the three instruments was 2.1, 2.7, 2.4 m, respectively for *E*, *A* and *B*. For the accepted value, we rely more on the buoy *B*, as *E* has large uncertainty because of its short record length (3 min, see Fig. 6). A direct inspection of the sequential *A* derived *H*_*s*_ values reveals a large variability out of which 2.7 was a maximum. As a conservative value (for our estimate of freak crest) we consider the measurement from *B*, i.e. *H*_*s*_ = 2.4 m. The peak period was 5.8 s. The top diagram in Fig. 6 reports the time evolution of the wave conditions in the short interval (1.5 hour) there shown. We have used the echo data because of their more frequent data.

As derived by the modeling hindcast (see the wave field in Fig. 5b), the 2*D*(*f, θ*) spectrum is provided in Fig. 2d. The corresponding *H*_*s*_ is 2.3 m. The condition shows a classical generative spectrum, with a progressive slight shift of the lower frequencies mean flow direction toward the coast due to refraction on the relative depth.

### Critical analysis

Lacking stereo-vision that may provide the 3D wave field, our estimates are based on a single view. Even using substantial know-how, these are obviously open to error. Aware of this, we have done a straightforward sensitivity analysis. For instance, in the second approach the boat positioning on the screen at various distances has been repeated three times. The differences have been a few percent. Similarly, the position of the crest has been allowed a 5% error. From the geometry in Fig. 4:

1 $$h=d\cdot \frac{\left(p-c\right)}{p}$$

and the *h* percent error depends on the ones of *c* and *p*. Considering an overall 10% error (see Fig. 4a), we still have a 6.4·0.90 = 5.7 m crest height. Pushing to 20% error, the crest would be 5.1 m high, still a very large (*C*_*crest*_/*H*_*s*_ = 2.1) value with respect to the 2.4 m local significant wave height, also considering^12^ possible non-linear effects.

### Instrumental data

The data at disposal (modelled wind and wave fields) suggest this was a normal mild bora storm. However, it was remarkable that a few minutes later the echo-sounder recorded another large wave, 4.92 m *H*_max_ on 2.3 m significant wave height. This second figure is even more remarkable thinking that at the time this system (see Fig. 6) recorded for only three minutes, corresponding to about 30 waves or so. The AWAC was in its inter-record interval.

The big wave was indeed recorded by the AWAC system as a 6.42 m *H*_*max*_,, a freak event according to definition (6.42/2.4 = 2.66), but not comparable to the webcam estimate of the crest. We interpret this as an indication of the non-linear character of the wave. We have a fully documented previous example in the heavy storm of 29 October 2018^13^, when, with *H*_*s*_ close to 6 m, a radar gauge recorded (at 2 Hz) a 9 m high crest of which there was no trace in the contemporary, otherwise perfectly consistent, AWAC record. The reason is that in strong conditions, with wave breaking, hence air bubbles in the upper layer, the AWAC system shifts to pressure recording. From this the surface elevation is derived on the basis of linear theory. This suggests a strong non-linearity of the big crest.

### Persistence

One of the remarkable aspects of “the wave” was its persistence in time and space, at least 20 sec after its already mature documented appearance and 200 m of progress with only a limited decrease of the crest height. This suggests different conditions from what reported by^2^ using the stereo system. While citing a large number of freak events when measuring in time and space, all their events were barely above the crest/height 1.25 limit, and, most of all, lasting a few seconds at most. On the contrary, the numerical work by^14^ shows clearly how, also in short crested waves, large freak events may have duration of ten or more periods. So the suggestion is that majestic events as the one here described must have a correspondingly larger and longer horizon and life.

## Data Availability

In order to get the internal parameters of the camera we have used the Camera Calibration Toolbox for Matlab, available at the following link: http://www.vision.caltech.edu/bouguetj/calib_doc/. The undistort freak wave image has been obtained by means of the TriDmetrix 2014 software, which is available at the following link: http://www.tridmetrix.com/.
